# Efficacy and Safety of Ipragliflozin in Japanese Patients With Type 2 Diabetes: Interim Outcome of the ASSIGN-K Study

**DOI:** 10.14740/jocmr2417w

**Published:** 2015-12-28

**Authors:** Takashi Iizuka, Kotaro Iemitsu, Masahiro Takihata, Masahiko Takai, Shigeru Nakajima, Nobuaki Minami, Shinichi Umezawa, Akira Kanamori, Hiroshi Takeda, Takehiro Kawata, Shogo Ito, Taisuke Kikuchi, Hikaru Amemiya, Mizuki Kaneshiro, Atsuko Mokubo, Tetsuo Takuma, Hideo Machimura, Keiji Tanaka, Taro Asakura, Akira Kubota, Sachio Aoyagi, Kazuhiko Hoshino, Masashi Ishikawa, Yoko Matsuzawa, Mitsuo Obana, Nobuo Sasai, Hideaki Kaneshige, Fuyuki Minagawa, Tatsuya Saito, Kazuaki Shinoda, Masaaki Miyakawa, Yasushi Tanaka, Yasuo Terauchi, Ikuro Matsuba

**Affiliations:** a Study Group of the Diabetes Committee, Kanagawa Physicians Association, Kanagawa, Japan; b Division of Metabolism and Endocrinology, Department of Internal Medicine, St. Marianna University School of Medicine, Kanagawa, Japan; c Department of Endocrinology and Diabetes, Yokohama City University Medical Center, Kanagawa, Japan

**Keywords:** Body composition, Type 2 diabetes mellitus, Sodium-glucose co-transporter 2 inhibitor, Ipragliflozin, Antidiabetic agent

## Abstract

**Background:**

Ipragliflozin is a sodium-glucose co-transporter 2 inhibitor that can improve glycemic control and reduce body weight and blood pressure in patients with type 2 diabetes mellitus (T2DM). We evaluated the efficacy and safety of ipragliflozin in the real-world clinical setting, with a focus on the changes of body composition up to 3 months of treatment.

**Methods:**

This was a prospective multicenter interventional trial. We investigated changes of the blood pressure, body composition, blood glucose, hemoglobin A1c (HbA1c), ketone bodies, lipids, and insulin after treatment with ipragliflozin (50 - 100 mg/day) for 12 weeks in Japanese patients with T2DM who showed poor glycemic control despite receiving diet and exercise therapy with or without oral antidiabetic drugs for more than 12 weeks.

**Results:**

Two hundred and fifty-seven subjects were included in the efficacy analysis up to 12 weeks of treatment and 301 subjects were included in the safety analysis. From baseline to 12 weeks, HbA1c showed a change of -0.68% (95% confidence interval (CI): -0.83, -0.53) and fasting blood glucose showed a change of -23.9 mg/dL (95% CI: -30.5, -17.2), with both parameters displaying a significant reduction (P < 0.001). The difference of body weight from baseline was -1.82 kg (95% CI: -2.14, -1.50), and it also showed significant reduction (P < 0.001). Analysis of body composition revealed that body fat changed by -1.46 kg (95% CI: -1.79, -1.14, P < 0.001) and body water changed by -0.37 kg (95% CI: -0.60, -0.14, P < 0.01). Laboratory tests demonstrated improvement of liver function and the lipid profile. Adverse events (AEs) occurred in 22.6% of the subjects, with frequent events being vulvovaginal candidiasis in 2.7% and cystitis in 2.0%. Serious AEs occurred in three subjects.

**Conclusions:**

In patients with T2DM, ipragliflozin improved glycemic control after 1 month of treatment and caused weight loss by reducing body fat more than body water.

## Introduction

Type 2 diabetes mellitus (T2DM) is a complex metabolic disease that is characterized by increased insulin resistance in the muscles and liver and/or decreased insulin secretion by pancreatic β cells [1]. Epidemiological investigation has shown that the determinants of T2DM are lifestyle and ethnicity, with lifestyle changes such as a high-fat diet and lack of exercise contributing to the development and progression of T2DM [2]. Most patients with T2DM are overweight or obese, and obesity is considered to be related to T2DM because obese persons have an elevated risk of developing this disease [3]. A meta-analysis conducted in Western countries showed that an increased body mass index (BMI) and waist circumference were significantly associated with the incidence of T2DM, and that the risk of T2DM was more than five times higher in obese men and more than 11 times higher in obese women compared with healthy adults [4]. On the other hand, genetic factors are important in non-obese patients with T2DM [5]. The Diabetes Prevention Studies (DPS) performed in the USA and Finland demonstrated that the risk of developing diabetes is reduced by weight loss (the chief determinant of risk reduction) through changes to dietary and exercise habits [6, 7]. In subjects who lost weight after modification of diet and exercise, the risk of developing diabetes in the future was reduced by about 25% per 1 kg of weight loss [8].

Although Japanese have a lower BMI than Westerners, the ERA JUMP study revealed that they show greater accumulation of visceral fat, particularly in the liver [9]. This suggests that it may be important for antidiabetic drugs to control the accumulation of visceral fat as well as regulating blood glucose. However, some of the current oral antidiabetic drugs like sulfonylureas (SUs) and thiazolidinediones promote weight gain [10], as do insulin formulations, making their use to improve glycemic control somewhat problematic in obese patients with T2DM.

Ipragliflozin is a sodium-glucose co-transporter 2 (SGLT2) inhibitor, which inhibits reabsorption of glucose in the proximal tubules of the kidneys to accelerate its urinary excretion and reduce the blood glucose level by a different mechanism from insulin and existing oral antidiabetic agents. This drug is expected to be used for patients with T2DM because it can improve glycemic control by a novel mechanism of action that results in calorie loss. In a study conducted by Kashiwagi et al, ipragliflozin (50 mg/day) was administered to Japanese patients with T2DM for 12 weeks, resulting in hemoglobin A1c (HbA1c) and body weight being significantly reduced from baseline by 0.79% and 1.81 kg, respectively [11], and its effect on glycemic control and weight in Japanese patients differed from the findings observed in Western patients [12]. It has also been reported that add-on treatment with an SGLT2 inhibitor is effective for improving glycemic control and preventing weight gain due to insulin therapy or dose escalation of insulin in obese T2DM patients using insulin with or without oral antidiabetic drugs [13, 14].

With respect to the safety of ipragliflozin, serious adverse events (AEs) reported up to 3 months after the start of treatment include urinary tract infection, hypoglycemia, dehydration, and skin lesions with an unknown mechanism. There is also concern about adverse drug reactions (ADRs), including hypoglycemia, in non-obese East Asian T2DM patients characterized by impaired insulin secretion [15]. Thus, ipragliflozin is characterized by achieving weight reduction, which does not occur with current antidiabetic drugs, as well as improving glucose and HbA1c. However, there have been no studies of its efficacy and safety during long-term administration or large-scale studies of ipragliflozin monotherapy, combination therapy with other antidiabetic drugs, or switching from other drugs to ipragliflozin.

In the present study, we performed interim analysis of the efficacy and safety of ipragliflozin, focusing on changes of body composition, based on data obtained at 12 weeks in the ASSIGN-K study, which is being conducted for “investigation of the appropriate use of SGLT2 inhibitors in clinical practice” by the Diabetes Committee of the Kanagawa Physicians Association.

## Patients and Methods

### Study design

This is a prospective multicenter interventional study that is designed to evaluate the efficacy and safety of ipragliflozin in patients with T2DM. Subjects were enrolled electronically at 33 centers in Japan from July 2014 to July 2015, and the study will continue until January 2017. After enrollment, ipragliflozin was administered at a dose of 50 - 100 mg once daily for 52 weeks. Study visits for observation/investigation were scheduled at baseline and in weeks 4, 12, 24, 36, and 52.

This study is being performed according to the Declaration of Helsinki and the Ethical Guideline for Clinical Research. We obtained approval of the study protocol and informed consent form from the Institutional Review Board of Meiwa Hospital (Dojin Kinen Kai Healthcare Corporation) on June 17, 2014 before the start of the study.

### Subjects

Male and female patients with T2DM were eligible for the study if they were aged 20 years or older, provided written informed consent, and had poor glycemic control (baseline HbA1c ≥ 6.0%; National Glycohemoglobin Standardization Program value) despite treatment for more than 12 weeks with diet and exercise therapy or diet and exercise combined with antidiabetic drugs. Patients who had a history of hypersensitivity to any component of the study drug were excluded, as were patients with a history of severe ketosis, diabetic coma, or precoma within past 6 months, postoperative patients or patients scheduled for surgery, and patients with severe infection, serious trauma, or severe renal dysfunction. Patients who were judged to be ineligible by the investigator were also excluded, as were pregnant or possibly pregnant women and nursing women.

### Efficacy endpoints

The efficacy endpoints included the changes from baseline of HbA1c, fasting blood glucose, postprandial blood glucose, weight, waist circumference, serum lipids, blood pressure, and body composition. These endpoints were assessed at the scheduled visits during the study. A bioimpedance analyzer (T-SCAN PLUS; Kobe Medicare Corporation, Kobe, Japan) was used for assessment of body composition, including the body weight, ideal body weight, BMI, body fat percentage, fat-free mass, body fat amount, muscle mass, mineral mass, protein mass, body water mass, intracellular water, and extracellular water.

### Safety endpoints

The safety endpoint included changes of the blood ketone level from baseline, as well as AEs and ADRs. AEs were coded according to the Medical Dictionary for Regulatory Activities (MedDRA/J version 18.0, Japanese Maintenance Organization, Tokyo, Japan).

### Statistical analysis

With respect to patient characteristics, continuous variables were summarized as the mean and standard deviation (SD), while categorical variables were reported as percentages. The endpoints at baseline, 4 weeks, and 12 weeks were assessed by multiple comparison using repeated measures analysis of variance. The level of significance was set at 5% (two-sided) and the two-sided 95% confidence interval (CI) was calculated. Statistical analysis was performed with SAS software (version 9.3).

## Results

### Patient characteristics

The safety analysis set included 301 subjects (139 men and 162 women) with data on AEs and ADRs throughout the study period. The efficacy analysis set included 257 subjects (123 men and 134 women) with measurements of HbA1c from baseline to 12 weeks, excluding for 33 patients who discontinued the study and 11 patients with missing data. Table 1 shows the characteristics of the 257 subjects in the efficacy analysis set. There were 123 men (47.9%) and 134 women (52.1%) with a mean age of 53.9 ± 10.4 years and mean T2DM duration of 9.6 ± 6.9 years. Their mean body weight was 80.29 ± 17.43 kg, BMI was 29.97 ± 5.39 kg/m^2^, HbA1c was 8.23±1.48%, fasting blood glucose was 158.5 ± 43.6 mg/dL (n = 110), and postprandial blood glucose was 199.1 ± 85.2 mg/dL (n = 93). The majority of the subjects (78%) used concomitant drugs, including dipeptidyl peptidase-4 (DPP-4) inhibitors in 154 subjects (77.0%), biguanides in 144 subjects (72.0%), SUs in 95 subjects (47.5%), insulin in 47 subjects (23.5%), thiazolidinediones in 45 subjects (22.5%), α-glucosidase inhibitors (α-GI) in 26 subjects (13.0%), and glinides in 11 subjects (5.5%). In addition, 94.2% of the subjects had complications, including dyslipidemia (70.0%), hypertension (56.0%), and fatty liver (50.2%) (data not shown).

**Table 1:** Profile of the Patients

	n%	Mean ± standard deviation
Sex		
Male	123 (47.9)	
Female	134 (52.1)	
Age, years	257	53.9 ± 10.4
Body weight, kg	257	80.29 ± 17.43
Body mass index, kg/m^2^	257	29.97 ± 5.39
Waist circumference, cm	253	101.19 ± 11.75
Duration of diabetes	257	9.6 ± 6.9
Hemoglobin A1c, %	257	8.23 ± 1.48
Fasting blood glucose , mg/dL	110	158.5 ± 43.6
Postprandial blood glucose, mg/dL	93	199.1 ± 85.2
Concomitant medications		
Dipeptidyl peptidase-4 inhibitor	154 (77.0)	
Biguanide	144 (72.0)	
Sulfonylurea	95 (47.5)	
Insulin	47 (23.5)	
Thiazolidinedione	45 (22.5)	
α-glucosidase inhibitor	26 (13.0)	
Glinide	11 (5.5)	

### Efficacy

The change of HbA1c from baseline after 4 and 12 weeks of treatment was -0.43% (95% CI: -0.51, -0.35) and -0.68% (95% CI: -0.83, -0.53), respectively, and there was a significant reduction at both times (P < 0.001). A significant reduction of HbA1c was also observed between 4 and 12 weeks of treatment (P < 0.001) (Table 2). At 4 weeks, the change of fasting blood glucose and postprandial blood glucose from baseline was -19.31 mg/dL (95% CI: -26.45, -12.17) and -38.14 mg/dL (95% CI: -55.49, -20.79), respectively, with a significant decrease in each parameter (P < 0.001). The reduction was maintained between 4 and 12 weeks of treatment, with no significant difference between these two times. Homeostatic model assessment of insulin resistance (HOMA-R) demonstrated that insulin resistance also decreased significantly from baseline to 4 weeks of treatment (P < 0.001). The improvement was subsequently maintained, with no significant difference being observed between 4 and 12 weeks of treatment. On the other hand, there was no significant change of HOMA-β, an indicator of insulin secretion, after 4 weeks of treatment, but a significant increase was observed at 12 weeks (P < 0.05).

**Table 2:** Summary of the Changes in Glycemic Control

	n	Baseline	4 weeks	12 weeks
Fasting blood glucose level, mg/dL	110	158 ± 43.6	139.2 ± 37.1***	134.6 ± 34.6***
vs. baseline			-19.3 ± 37.8	-23.9 ± 35.2
95% CI			-26.4, -12.2	-30.5, -17.2
Postprandial blood glucose level, mg/dL	93	199.1 ± 1.49	161.0 ± 60.0***	54.9 ± 60.5***
vs. baseline			-38.1 ± 84.3	-44.2 ± 77.0
95% CI			-55.5, -20.8	-60.1, -28.4
Hemoglobin A1c, %	257	8.23 ± 1.49	7.81 ± 1.24***	7.55 ± 1.28***†††
vs. baseline			-0.43 ± 0.64	-0.68 ± 1.23
95% CI			-0.51, -0.35	-0.83, -0.53
HOMA-R	55	4.62 ± 3.68	3.35 ± 2.43**	3.36 ± 2.45***
vs. baseline			-1.27 ± 2.73	-1.26 ± 1.93
95% CI			-2.01, -0.53	-1.78, -0.73
HOMA-β	55	46.2 ± 43.7	61.0 ± 87.3	54.9 ± 45.6*
vs. baseline			14.7 ± 83.7	8.6 ± 26.0
95% CI			-7.9, -37.4	1.6, 15.7
Insulin, μU/mL	73	12.79 ± 8.90	11.79 ± 9.32	13.89 ± 20.64
vs. baseline			-1.00 ± 7.42	1.10 ± 16.46
95% CI			-2.73, -0.74	-2.74, 4.94

ANOVA vs. 0 weeks, ***P < 0.001, **P < 0.01, *P < 0.05; 4 weeks vs. 12 weeks, †††P < 0.001, ††P < 0.01, †P < 0.05. CI: confidence interval; HOMA: homeostatic model assessment.

Changes of blood pressure and fasting serum lipids are summarized in Table 3. Both systolic and diastolic blood pressures were significantly reduced after 4 weeks of treatment (P < 0.001) and the reduction was maintained at 12 weeks. With respect to lipids, triglycerides showed a significant decrease at 12 weeks (P < 0.05), while high-density lipoprotein (HDL) cholesterol was significantly increased (P < 0.001). Significant changes of total cholesterol and non-HDL cholesterol were also observed at 4 weeks, but not at 12 weeks. There were no significant changes of non-esterified fatty acids and low-density lipoprotein (LDL) cholesterol.

**Table 3:** Summary of the Changes of Blood Pressure and Fasting Serum Lipids

	n	Baseline	4 weeks	12 weeks
Systolic blood pressure, mm Hg	254	131.3 ± 15.9	126.9 ± 15.9***	127.3 ± 15.6***
vs. baseline			-4.3 ± 14.1	-4.0 ± 14.6
95% CI			-6.1, -2.6	-5.8, -2.2
Diastolic blood pressure, mm Hg	254	78.3 ± 10.6	76.4 ± 10.2**	76.3 ± 11.0**
vs. baseline			-1.9 ± 9.4	-2.1 ± 10.3
95% CI			-3.1, -0.8	-3.3, -0.8
Free fatty acids, μEq/L	218	630.7 ± 303.9	664 ± 308.0	624.3 ± 338.0
vs. baseline			33.3 ± 325.6	-6.5 ± 360.3
95% CI			-10.2, 76.8	-54.6, 41.6
Triglycerides, mg/dL	254	203.0 ± 164.9	182.7 ± 113.8	180.8 ± 129.1*
vs. baseline			-20.3 ± 141.2	-22.3 ± 149.1
95% CI			-37.8, -2.9	-40.7, -3.8
LDL cholesterol, mg/dL	208	114.2 ± 32.7	111.5 ± 34.0	112.5 ± 31.1
vs. baseline			-2.8 ± 24.6	-1.7 ± 25.2
95% CI			-6.1, 0.6	-5.2, 1.7
HDL cholesterol, mg/dL	252	50.6 ± 13.1	50.9 ± 13.4	53.3 ± 16.1**††
vs. baseline			0.3 ± 8.7	2.7 ± 12.9
95% CI			-0.8, 1.4	1.1, 4.3
Total cholesterol, mg/dL	243	201.8 ± 40.2	196.9 ± 38.9*	200.3 ± 37.9
vs. baseline			-4.9 ± 28.3	-1.5 ± 29.6
95% CI			-8.4, -1.3	-5.3, 2.2
Non-HDL cholesterol, mg/dL	241	151.1 ± 40.9	146.2 ± 40.7*	147.0 ± 38.6
vs. baseline			-4.9 ± 28.0	-4.1 ± 30.8
95% CI			-8.4, -1.3	-8.0, -0.2

ANOVA vs. 0 weeks, ***P < 0.001, **P < 0.01, *P < 0.05; 4 weeks vs. 12 weeks, †††P < 0.001, ††P < 0.01, †P < 0.05. CI: confidence interval; LDL: low-density lipoprotein; HDL: high-density lipoprotein.

Table 4 shows the changes of liver function and renal function tests. Alkaline phosphatase, alanine aminotransferase (ALT), aspartate transaminase (AST), and lactate dehydrogenase were almost within the normal range at baseline, but significant reduction was observed after 4 and 12 weeks of treatment. ALT and AST showed a significant difference between 4 and 12 weeks of treatment (P < 0.001). At baseline, gamma-glutamyl transpeptidase (γ-GTP) was higher than normal and it remained high after 4 and 12 weeks of treatment, but significant reduction from baseline was observed at each time (P < 0.001). Blood urea nitrogen (BUN) and creatinine increased significantly after the start of treatment, while uric acid displayed a significant decrease from 4 weeks of treatment (P < 0.001). Hematocrit was significantly increased after 4 and 12 weeks of treatment (both P < 0.001), and a significant difference was also observed between 4 and 12 weeks (P < 0.001). Sodium (Na) showed a significant increase after 4 weeks of treatment, but there was no increase at 12 weeks. Potassium (K) demonstrated a significant increase from baseline to 12 weeks of treatment (P < 0.001), and a significant increase was also observed between 4 and 12 weeks (P < 0.001).

**Table 4:** Summary of the Changes in Laboratory Values

	n	Baseline	4 weeks	12 weeks
ALP, IU/L	246	239.9 ± 88.1	228.3 ± 75.5***	230.1 ± 72.4**
vs. baseline			-11.7 ± 42.9	-9.9 ± 47.5
95% CI			-17.0, -6.3	-15.8, -3.9
ALT, IU/L	251	42.9 ± 32.4	39.6 ± 31.8*	35.1 ± 24.0***†††
vs. baseline			-3.3 ± 18.6	-7.7 ± 24.1
95% CI			-5.6, -0.9	-10.7, -4.8
AST, IU/L	252	35.3 ± 27.8	33.1 ± 26.5*	29.1 ± 18.1***†††
vs. baseline			-2.2 ± 12.1	-6.2 ± 18.7
95% CI			-3.7, -0.7	-8.5, -3.9
γ-GTP, IU/L	251	65.6 ± 74.8	53.9 ± 61.4***	47.7 ± 48.6***†
vs. baseline			-11.7 ± 32.5	-17.9 ± 54.8
95% CI			-15.7, -7.6	-24.7, -11.1
LDH, IU/L	242	203.9 ± 43.4	196.6 ± 39.6***	194.1 ± 38.3***
vs. baseline			-7.3 ± 29.9	-9.9 ± 36.8
95% CI			-11.1, -3.5	-14.5, -5.2
BUN, mg/dL	250	14.1 ± 4.4	14.7 ± 4.4*	15.1 ± 4.3***
vs. baseline			0.6 ± 3.3	1.0 ± 3.6
95% CI			0.2, 1.0	0.6, 1.5
Uric acid, mg/dL	245	5.6 ± 1.6	5.2 ± 1.9***	5.0 ± 1.3***
vs. baseline			-0.4 ± 1.7	-0.6 ± 1.5
95% CI			-0.6, -0.2	-0.7, -0.4
Serum creatinine, mg/dL	250	0.70 ± 0.19	0.73 ± 0.20***	0.71 ± 0.21†
vs. baseline			0.02 ± 0.08	0.01 ± 0.10
95% CI			0.01, 0.03	-0.01, 0.02
Na^+^, mEq/L	247	140.6 ± 2.4	141.0 ± 2.2*	140.8 ± 2.1
vs. baseline			0.3 ± 2.1	0.2 ± 2.3
95% CI			0.1, 0.6	-0.1, 0.4
K^+^, mEq/L	245	4.1 ± 0.5	4.1 ± 0.5	4.3 ± 0.5***†††
vs. baseline			0.1 ± 0.4	0.2 ± 0.5
95% CI			0.0, 0.1	0.2, 0.3
Cl^-^, mEq/L	245	102.4 ± 3.1	102.9 ± 3.0**	102.7 ± 2.7
vs. baseline			0.5 ± 2.4	0.2 ± 2.5
95% CI			0.2, 0.8	-0.1, 0.6
P, mg/dL	234	3.3 ± 0.6	3.5 ± 0.6***	3.4 ± 0.5*
vs. baseline			0.1 ± 0.5	0.1 ± 0.5
95% CI			0.1, 0.2	0.0, 0.2
Blood ketone bodies, mmol/L	197	0.28 ± 0.46	0.32 ± 0.48	0.28 ± 0.35
vs. baseline			0.05 ± 0.41	0.01 ± 0.56
95% CI			-0.01, 0.10	-0.07, 0.09
Hematocrit, %	249	43.1 ± 4.2	44.0 ± 5.1***	45.0 ± 4.6***†††
vs. baseline			0.9 ± 3.3	1.9 ± 3.4
95% CI			0.5, 1.3	1.5, 2.3

ANOVA vs. 0 weeks, ***P < 0.001, **P < 0.01, *P < 0.05; 4 weeks vs. 12 weeks; †††P < 0.001, ††P < 0.01, †P < 0.05. CI: confidence interval; ALP: alkaline phosphatase; ALT: alanine aminotransferase; AST: aspartate transaminase; γ-GTP: gamma-glutamyl transpeptidase; LDH: lactate dehydrogenase; BUN: blood urea nitrogen.

**Figure 1:**
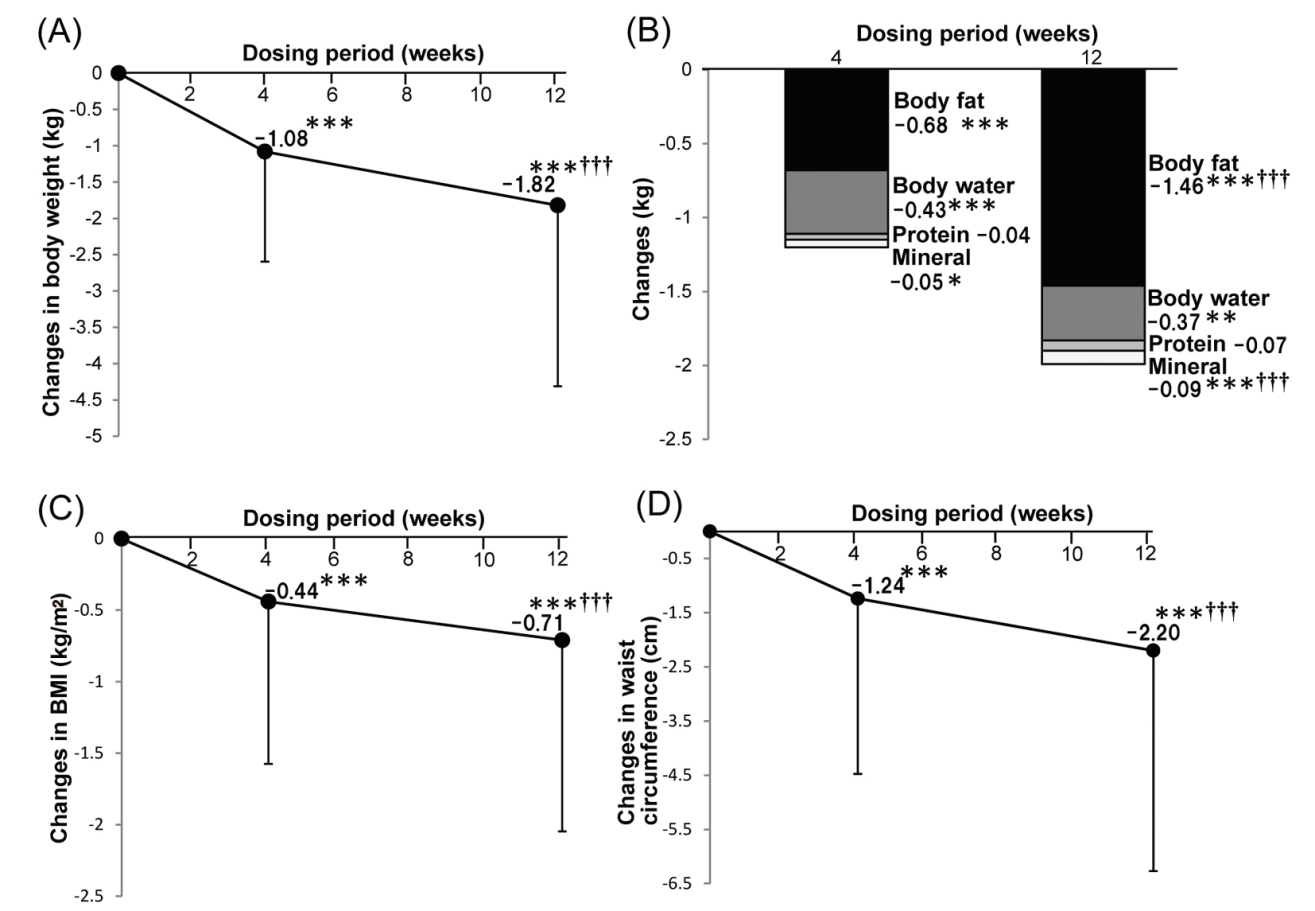
Effects of ipragliflozin therapy on body composition. Changes of body weight after ipragliflozin treatment (n = 240) (A). Changes of body composition parameters after ipragliflozin treatment (n = 239) (B). Changes of body mass index after ipragliflozin treatment (n = 241) (C). Changes of waist circumference after ipragliflozin treatment (n = 250) (D). Data are shown as the mean ± standard deviation. Significant differences after 4 and 12 weeks of treatment compared with baseline: ***P < 0.001, **P < 0.01, *P < 0.05. Significant differences between 4 and 12 weeks of treatment: †††P < 0.001, ††P < 0.01, †P < 0.05.

The change of body weight from baseline to week 4 of treatment was -1.08 kg (95% CI: -1.27, -0.89) and the change to 12 weeks was -1.82 kg (95% CI: -2.14, -1.50), with significant reduction being noted (P < 0.001). There was also a significant decrease in weight between 4 and 12 weeks of treatment (P < 0.001) (Fig. 1A). Body composition analysis revealed that the change of body fat from baseline to 4 weeks of treatment was -0.68 kg (95% CI: -0.95, -0.40) and the change to 12 weeks was -1.46 kg (95% CI: -1.79, -1.14), with a significant reduction being noted at each time (P < 0.001) (Fig. 1B). Body water mass was the second largest factor contributing to the change of body weight after body fat. The change of body water mass was -0.43 kg (95% CI: -0.62, -0.24, P < 0.001) after 4 weeks of treatment and -0.37 kg (95% CI: -0.60, -0.14, P < 0.01) at 12 weeks, showing a significant reduction at each time (Fig. 1B). Among body water components, the changes of extracellular water and intracellular water from baseline were respectively -0.41 kg (95% CI: -0.57, -0.25, P < 0.0001) and 0.003 kg (95% CI: -0.215, 0.222, P > 0.05) after 4 weeks of treatment, while the changes were respectively -0.60 kg (95% CI: -0.79, -0.40, P < 0.0001) and 0.262 kg (95% CI: 0.032, 0.492, P > 0.05) at 12 weeks. Thus, there were no significant changes of intracellular water and most of the change in body water mass was related to fluctuation of extracellular water. However, there was no significant difference of extracellular water between 4 and 12 weeks of treatment. In addition, a significant reduction of mineral mass was observed from 4 weeks of treatment (P < 0.05 at 4 weeks and P < 0.001 at 12 weeks), but there were no significant changes of protein mass.

The changes of BMI and waist circumference from baseline were respectively -0.44 kg/m^2^ (95% CI: -0.58, -0.30) and -1.24 cm (95% CI: -1.65, -0.84) after 4 weeks of treatment, while the respective changes were -0.71 kg/m^2^ (95% CI: -0.88, -0.54) and -2.20 cm (95% CI: -2.71, -1.69) at 12 weeks, with a significant reduction being seen at each time (P < 0.0001, Fig. 1C, D).

### Safety

Blood ketone levels were higher after 4 and 12 weeks of treatment, increasing from around 0.28 ± 0.46 mmol/L at baseline, but no significant changes were observed.

AEs occurred in 68 subjects (22.6%) (Table 5). The main AEs included vulvovaginal candidiasis in eight subjects (2.7%); cystitis in six subjects (2.0%); influenza in six subjects (2.0%); genital pruritus and upper respiratory tract infection in five subjects (1.7%) each; eczema and nasopharyngitis in four subjects (1.3%) each; nausea, drug eruption, and constipation in three subjects (1.0%) each; hypoglycemia in two subjects (0.7%); and ketosis, urinary tract infection, unstable angina, and dehydration in one subject (0.3%) each. Three subjects (one each with ketosis, unstable angina, and urinary tract infection) were judged to have serious AEs, but all of these events resolved. ADRs were seen in 34 subjects (11.3%), including vulvovaginal candidiasis in eight subjects (2.7%); cystitis in six subjects (2.0%); genital pruritus in five subjects (1.7%); drug eruption in three subjects (1.0%); eczema in two subjects (0.7%); and hypoglycemia, ketosis, dehydration, vulvar discomfort, unstable angina, urinary tract infection, nausea, and constipation in one subject (1.0%) each.

**Table 5:** Summary of Adverse Events

	Number of subjects	Incidence (%)
All adverse events	68	22.6
Adverse events resulting in discontinuation of treatment	21	7.0
Adverse reactions	34	11.3
Adverse events resulting in hospitalization	3	1.0
Serious adverse events	3	1.0
Deaths	0	0
Main adverse events		
Vulvovaginal candidiasis	8	2.7
Cystitis	6	2.0
Influenza	6	2.0
Genital pruritus	5	1.7
Upper respiratory tract infection	5	1.7
Eczema	4	1.3
Nasopharyngitis	4	1.3
Nausea	3	1.0
Drug eruption	3	1.0
Constipation	3	1.0
Hypoglycemia	2	0.7
Ketosis†	1	0.3
Urinary tract infection†	1	0.3
Unstable angina†	1	0.3
Dehydration	1	0.3
Vulvovaginal discomfort	1	0.3

†Serious adverse events.

## Discussion

In this study based on daily clinical practice, there was improvement of blood glucose, body weight, blood pressure, and various laboratory values when ipragliflozin was administered alone or in combination with other oral antidiabetic drugs for 12 weeks to T2DM patients who had poor glycemic control despite receiving diet and exercise therapy with or without antidiabetic drugs. AEs occurred in 22.6% of the subjects and ADRs occurred in 11.3%, with serious AEs being reported in three subjects.

In a previous study by Kashiwagi et al, ipragliflozin was administered at 50 mg/day for 12 weeks to Japanese patients with T2DM, resulting in a change of HbA1c by -0.79% and fasting blood glucose by -34.1 mg/dL after treatment [12]. Both parameters showed greater improvement in that study than in the present investigation. Differences of baseline patient characteristics, including fasting blood glucose, HbA1c, and the duration of diabetes, may have contributed to these differing results. In addition, 28 patients switched from other treatments to ipragliflozin at the start of this study and they had higher HbA1c levels, which could also have influenced our results. It was previously reported that insulin resistance [16] and pancreatic function [17] were improved by treatment with ipragliflozin. Similarly, insulin resistance was reduced after 4 weeks of treatment in this study and insulin secretion was improved from 12 weeks. It is thought that pancreatic function was improved by the indirect actions of this SGLT2 inhibitor instead of its direct actions, because SGLT2 expression is not seen in the pancreas. Interestingly, it has been reported that β-cell function was maintained and β cell numbers were increased in db/db-SGLT2^-/-^ mice despite feeding with a high-fat diet for 4 weeks [18]. Therefore, the effects of ipragliflozin on β-cell function should be investigated further in the future.

In this study, there was similar improvement of body weight and waist circumference as reported in previous studies of ipragliflozin [12, 17]. However, there was a significant difference of both variables between 4 and 12 weeks of treatment (P < 0.001), as was also observed for HbA1c in this study, suggesting that a plateau for the effects of ipragliflozin was not reached before 12 weeks. Therefore, a longer study will be required to fully evaluate the efficacy of ipragliflozin. Significant reduction of both systolic and diastolic blood pressures was observed after 4 weeks of treatment (P < 0.001), which was considered to be due to osmotic diuresis and weight loss caused by ipragliflozin. Among the drugs used to treat T2DM, SUs, insulin formulations, and thiazolidinediones can cause weight gain, while biguanides, DPP-4 inhibitors, and α-GIs have no effect on body weight, and only glucagon-like peptide 1 analogs reduce weight [11]. Although the subjects of the present study received ipragliflozin in combination with oral antidiabetic drugs that can cause weight gain, most of them (87.8%) actually showed significant weight reduction, a potentially important clinical finding.

Body composition analysis revealed that about 60% of the weight loss achieved by the subjects after 4 weeks of treatment was due to reduction of body fat and about 39% was due to reduction of water (mainly extracellular water). On the other hand, only body fat showed further reduction after 12 weeks of treatment (-1.46 ± 2.57 kg, 4 weeks vs. 12 weeks, P < 0.001), while there was no further change of body water. These results were similar to those reported by Bolinder et al, who investigated the effect of dapagliflozin on body weight in T2DM patients by using dual-energy X-ray absorptiometry and magnetic resonance imaging [19]. Body fat reduction was presumably related to calorie loss (generally 200 - 300 kcal/day), while osmotic diuresis associated with urinary glucose excretion led to body water reduction during the early treatment period, and both of these changes contributed to early weight loss. Subsequent weight loss up to 12 weeks of treatment was mainly due to the continuing reduction of body fat. The hematocrit was increased in the present study, as also reported by Kashiwagi et al [12], and this was possibly associated with the reduction of body water. However, there were also significant increases in hemoglobin and the red cell count that are more difficult to explain, and further investigations of this issue should be undertaken in the future. In addition, there was a significant increase in BUN and a slight change of creatinine that were thought to be secondary to reduction of body water, but this also needs future investigation.

A previous study based on dual-energy X-ray absorptiometry revealed greater reduction of visceral fat than subcutaneous fat with dapagliflozin treatment [19]. The bioimpedance analyzer used in the present study can clearly distinguish between changes of body fat and body water, but cannot separate subcutaneous fat from visceral fat. In this study, waist circumference was also significantly reduced by 12 weeks of treatment, and this change was considered to mainly reflect the reduction of visceral fat. It has been reported that insulin sensitivity can be reduced by stimulating the secretion of free fatty acids, tumor necrosis factor α, and resistin from enlarged adipocytes [20], and there is a correlation between insulin resistance and the visceral fat load [21, 22]. In this study, a very weak positive correlation was also observed between changes of insulin resistance and body fat after 12 weeks of treatment (Pearson’s correlation analysis, r = 0.288, P < 0.05, n = 70). Further investigation of this correlation over a longer period may be required.

There was more marked improvement of clinical laboratory parameters, including ALT and AST, after 12 weeks of treatment in the present study than reported by Kashiwagi et al [12]. Because it was recently reported that weight loss was associated with reduction of liver weight and the hepatic triglyceride content after SGLT2 therapy in rodent diabetes models [23], the weight loss seen in this study could be associated with a mechanism other than improvement of visceral fat by calorie loss. With respect to serum lipids, HDL cholesterol was increased, triglycerides were reduced, and LDL cholesterol was unchanged after ipragliflozin treatment, suggesting that this drug has an antiarteriosclerotic effect. It is thought that HDL cholesterol was increased because of the reduction of visceral fat, while triglycerides were reduced by acceleration of gluconeogenesis, improvement of glycemic control, and weight loss.

With regard to the safety of ipragliflozin, AEs were reported in 68 subjects (22.6%) and ADRs were reported in 34 subjects (11.3%) up to 12 weeks of treatment. However, there were no significant changes of ketone bodies, one of the important safety endpoints. The most common events were genital and urologic diseases (4.7%), followed by skin conditions (2.3%). There were only three serious AEs (ketosis, unstable angina, and urinary tract infection reported in one subject each) and these events all resolved. Ketosis was detected in a patient who continued treatment while acutely ill with vomiting and diarrhea, suggesting that it is important to give precise instructions to all patients about suspending treatment during acute illness. In addition, AEs due to dehydration are more likely to occur near the start of treatment, suggesting that patients should be advised to maintain an adequate water intake before the start of treatment.

### Conclusions

The present study was an interim analysis of patients with data available up to 12 weeks of treatment in the ongoing ASSIGN-K study of ipragliflozin for T2DM. There was improvement of blood glucose, body weight, blood pressure, liver function, and the lipid profile after treatment with ipragliflozin alone or in combination with other oral antidiabetic drugs. Body composition analysis revealed a reduction of body fat and extracellular water after 1 month of treatment, but longer-term investigation will be required to fully evaluate the efficacy and safety of ipragliflozin.
