# Correction: Efficacy and Safety of Ipragliflozin in Japanese Patients With Type 2 Diabetes: Interim Outcome of the ASSIGN-K Study

**DOI:** 10.14740/jocmr2417wc1

**Published:** 2016-01-26

**Authors:** Takashi Iizuka, Kotaro Iemitsu, Masahiro Takihata, Masahiko Takai, Shigeru Nakajima, Nobuaki Minami, Shinichi Umezawa, Akira Kanamori, Hiroshi Takeda, Takehiro Kawata, Shogo Ito, Taisuke Kikuchi, Hikaru Amemiya, Mizuki Kaneshiro, Atsuko Mokubo, Tetsuo Takuma, Hideo Machimura, Keiji Tanaka, Taro Asakura, Akira Kubota, Sachio Aoyagi, Kazuhiko Hoshino, Masashi Ishikawa, Yoko Matsuzawa, Mitsuo Obana, Nobuo Sasai, Hideaki Kaneshige, Fuyuki Minagawa, Tatsuya Saito, Kazuaki Shinoda, Masaaki Miyakawa, Yasushi Tanaka, Yasuo Terauchi, Ikuro Matsuba

**Affiliations:** a Study Group of the Diabetes Committee, Kanagawa Physicians Association, Kanagawa, Japan; b Division of Metabolism and Endocrinology, Department of Internal Medicine, St. Marianna University School of Medicine, Kanagawa, Japan; c Department of Endocrinology and Diabetes, Yokohama City University Medical Center, Kanagawa, Japan

Corrections to article “Efficacy and Safety of Ipragliflozin in Japanese Patients With Type 2 Diabetes: Interim Outcome of the ASSIGN-K Study”, by Takashi Iizuka et al, published in Vol. 8, No. 2, 2016, p116-125, doi: http://dx.doi.org/10.14740/jocmr2417w

There were some errors in Table 2, the authors would like to make the following corrections.

The baseline postprandial blood glucose level (mg/dL) should read 199.1 ± 85.2, instead of 199.1 ± 1.49.

The 12 weeks postprandial blood glucose level (mg/dL) should read 154.9 ± 60.5, instead of 54.9 ± 60.5.

